# Long-term functional outcomes and surgical retreatment after thulium laser enucleation of the prostate: A 10-year follow-up study

**DOI:** 10.1590/S1677-5538.IBJU.2024.0039

**Published:** 2024-03-10

**Authors:** Celeste Manfredi, Luigi Napolitano, Francesco Ditonno, Giovanni Maria Fusco, Carmelo Quattrone, Marco De Sio, Luca Romis, Filippo Riccardo, Maria Rosaria Nugnes, Giovanni Di Lauro, Francesco Trama

**Affiliations:** 1 University of Campania "Luigi Vanvitelli" Unit of Urology, Child and General and Specialized Surgery Department of Woman Naples Italy Department of Woman - Unit of Urology, Child and General and Specialized Surgery, University of Campania "Luigi Vanvitelli", Naples, Italy;; 2 University of Naples "Federico II" Urology Unit Department of Neurosciences, Reproductive Sciences and Odontostomatology Naples Italy Department of Neurosciences, Reproductive Sciences and Odontostomatology, Urology Unit, University of Naples "Federico II", Naples, Italy;; 3 University of Verona Department of Urology Verona Italy Department of Urology, University of Verona, Azienda Ospedaliera Universitaria Integrata, Verona, Italy;; 4 Rush University Medical Center Department of Urology Chicago IL USA Department of Urology, Rush University Medical Center, Chicago, IL, USA;; 5 Urology Complex Unit-ASL Napoli 2 Nord "Santa Maria delle Grazie" Hospital Pozzuoli Italy Urology Complex Unit-ASL Napoli 2 Nord "Santa Maria delle Grazie" Hospital, Pozzuoli, Italy

**Keywords:** Prostatic Hyperplasia, Surgical Procedures, Operative, Thulium

## Abstract

**Background:**

To evaluate the 10-year functional outcomes (primary) and frequency and predictors of BPH surgical retreatment (secondary) after ThuLEP.

**Materials and Methods:**

A single-center retrospective analysis of consecutive patients undergoing ThuLEP between 2010 and 2013 was performed. Inclusion criteria were: age ≥ 40 years, prostate volume (PV) ≥ 80 mL, International Prostate Symptom Score (IPSS)-Total score ≥ 8 points. IPSS-Total score was the primary outcome, and BPH surgical retreatment rate was the secondary outcome. Paired t-test, McNemar test, and Wilcoxon signed-rank test were used to compare variables. Logistic regression analysis was performed to evaluate predictors of surgical retreatment.

**Results:**

A total of 410 patients with a mean ±SD age of 63.9 ± 9.7 years and a PV of 115.6 ± 28.6 mL were included. Mean ±SD follow-up was 108.2 ± 29.6 months. IPSS-Total score was significantly improved at 1 year compared to baseline (23.3 ± 4.7 vs. 10.3 ± 3.8; p<0.001). It was similar after 5 years (10.5 ± 3.6 vs. 10.7 ± 5.0; p=0.161), with a significant worsening at 10 years (10.3 ±4.8 vs. 13.8 ±4.5; p=0.042) but remaining statistically and clinically better than baseline (13.8 ±4.5 vs. 22.1 ±4.3; p<0.001). After 10 years, 21 (5.9%) patients had undergone BPH reoperation. Baseline PV (adjusted OR 1.27, 95% CI 1.09-1.41; p<0.001) and time from BPH surgery (adjusted OR 1.32, 95% CI 1.15-1.43; p<0.001) were predictors of BPH surgical retreatment.

**Conclusions:**

ThuLEP is associated with optimal functional outcomes and a low frequency of BPH surgical retreatment in the long-term. Baseline PV and time from surgery were predictors of BPH reoperation.

## INTRODUCTION

Benign prostatic hyperplasia (BPH) is a major cause of lower urinary tract symptoms (LUTS). Surgery should be offered to men with moderate-to-severe BPH-related LUTS that are unresponsive to medical treatment or have complications resulting from lower urinary tract obstruction (1). Transurethral resection of the prostate (TURP) remains the gold standard surgical treatment for BPH-related LUTS in patients with medium-sized prostates. Recently, we have witnessed the spread of endoscopic enucleation. Laser prostatic enucleation has become the new gold standard for the surgical treatment of BPH-related LUTS in men with large prostates, and it is considered a valid alternative to TURP for medium-sized glands. Consequently, laser enucleation appears to be a reliable procedure for treating BPH-related LUTS, regardless of prostate size (2).

Among the laser enucleation techniques, Holmium Laser Enucleation of the Prostate (HoLEP) and Thulium Laser Enucleation of the Prostate (ThuLEP) are the most commonly used. Currently, more evidence is available for HoLEP; however, a clear superiority of this technique over ThuLEP has not been demonstrated. Traditional thulium laser supplies continuous energy with a 2013 nm wavelength and a 0.25 mm penetration depth, providing excellent hemostasis; these characteristics make it an excellent technology for the enucleation of prostate tissue (3). Herrmann et al. first described ThuLEP as an enucleation technique in 2010 (4). The relatively recent introduction of ThuLEP explains the limited evidence available regarding its long-term outcomes. In particular, after surgery, prostatic tissue may reform over the years; as a result, LUTS may worsen, leading to the need to restart BPH medications and, in some cases, surgical retreatment (5).

The primary aim of this study was to evaluate the 10-year functional outcomes of ThuLEP. The secondary objective was to assess the frequency and predictors of BPH surgical retreatment 10 years after ThuLEP.

## MATERIALS AND METHODS

### Study design and Ethical details

We performed a retrospective analysis of a prospectively maintained database including patients undergoing ThuLEP between 2010 and 2013 at Santa Maria delle Grazie Hospital (Pozzuoli, Naples, Italy). The study obtained exempt status after being reviewed by the local Ethics Committee.

This research was conducted in accordance with the Declaration of Helsinki on ethical principles for medical research involving human subjects. All patients provided written informed consent for the inclusion of their data in the database and for their use for scientific research purposes.

### Study population

Consecutive patients undergoing ThuLEP were included in this study. Surgery was indicated in men with moderate-to-severe BPH-related LUTS, despite the maximum possible/desired medical therapy for BPH. Refractory acute urinary retention with an indwelling bladder catheter was an additional indication for surgery.

The inclusion criteria were age ≥ 40 years, prostate volume (PV) ≥ 80 mL, International Prostate Symptom Score (IPSS)-Total score ≥ 8 points (or indwelling bladder catheter) despite BPH medical treatment (6). The exclusion criteria were previous prostatic surgery, neurogenic bladder, suspicion/history of prostate cancer, history of bladder cancer, history of pelvic radiotherapy, interstitial cystitis, previous urethral stricture, chronic prostatitis, recurrent urinary tract infections, and bladder stones. Patients with missing baseline data were excluded from the study. The initial 20 cases of ThuLEP were excluded to limit the impact of the learning curve on outcomes (7).

### Patient evaluation and Outcomes

Before ThuLEP, patients underwent a thorough clinical evaluation and the following parameters were recorded: age, body mass index (BMI), diabetes mellitus, digital rectal examination (DRE), PV (by transrectal ultrasound), prostate-specific antigen (PSA), presence of an indwelling bladder catheter, BPH medications, maximum urinary flow rate (Qmax), average urinary flow rate (Qave), post-void residual volume (PVR), and IPSS.

The patients underwent an in-person follow-up visit at 1, 5, and 10 years after ThuLEP. Each follow-up visit was requested and scheduled by the secretariat via telephone call to the patient and was performed by an expert urologist. PV (by transrectal ultrasound), IPSS-Total score, IPSS-QoL, Qmax, Qave, and PVR were evaluated at each follow-up. The Minimal Clinically Important Difference (MCID) for IPSS-Total score, IPSS-QoL, and Qmax were also assessed, considering −3 points, −0.5 points, and +2 mL/s as the respective cut-offs (clinical improvement) (8). Patient Global Impression of Improvement (PGI-I) of urinary function compared to baseline was collected at each follow-up visit (9). Men undergoing BPH medical therapy or BPH surgical retreatment after ThuLEP for worsening LUTS were recorded and not excluded from the analysis of functional outcomes. BPH surgical retreatment was defined as any surgical procedure performed on the prostate after ThuLEP to improve the patient's urinary outcomes. Surgery for hemostasis of the lower urinary tract, urethral surgery, reconstructive surgery of the bladder neck, and urinary incontinence surgery were excluded from the definition of BPH surgical retreatment. The number of patients lost at each follow-up interval with related causes were recorded. For the calculation of the mean follow-up, patients undergoing surgical retreatment were considered up to this event.

The IPSS-total score was chosen as the primary outcome, while BPH surgical retreatment rate was selected as the secondary outcome.

### Surgical details

All surgical procedures were performed by a single experienced surgeon (GDL) with the same technique and devices.

A RevoLix™ Thulium Laser (LISA Laser Products GmbH, Katlenburg-Lindau, Germany) was used for all ThuLEPs. The laser energy was delivered via a reusable 550 μm laser fiber (RigiFib™, Laser Products GmbH, Katlenburg-Lindau, Germany) directed with a 26 F continuous-flow resectoscope (Karl Stortz GmbH, Tuttlingen, Germany). The power settings of the laser device for the enucleation and coagulation of the prostatic tissue were 70 W and 30 W, respectively. Morcellation was performed using a Piranha™ Morcellation System (Richard Wolf GmbH, Knitdlingen, Germany) set at a speed of 1,500 rpm.

The procedure was divided into three successive surgical phases: laser enucleation of the prostatic lobes, laser coagulation of the capsule, and mechanical morcellation of the enucleated tissue. The enucleation was represented by the 3-lobe technique previously proposed by Herrmann et al.: 1) inverted U-shaped incision proximal to the verumontanum deepened up to the surgical capsule; 2) incision at 5 o'clock and 7 o'clock from the bladder neck to the inverted U-shaped incision and removal of the median lobe; 3) removal of the lateral lobes one at a time (starting from the left) by incision of the mucosa and opening of the plane between the hyperplastic tissue and the surgical capsule (4). In case of bleeding not adequately controlled with laser coagulation alone, monopolar electrical coagulation was also used.

At the end of each surgical procedure, a 22 Ch 3-way Dufour tip Foley catheter was placed and continuous low-flow bladder irrigation with saline solution was applied. All BPH medications were suspended from the day of surgery.

### Statistics

The continuous variables were described as means and standard deviations (SDs), while the categorical variables were expressed as absolute and relative frequencies. The Kolmogorov-Smirnov test was applied as a normality test (10). The paired t-test, McNemar test, and Wilcoxon signed-rank test were used to compare continuous, dichotomous, and ordinal variables, respectively, at different follow-up times (11). The Kaplan-Meier method was used to analyze time to BPH surgical retreatment (12). A p-value < 0.05 was arbitrarily set to indicate statistical significance. Logistic regression analysis was performed to evaluate predictors of BPH surgical retreatment 10 years after ThuLEP. Age, baseline PV, BPH medical retreatment, and time from BPH surgery were selected *a priori* as independent variables for multivariable analysis. The results of the logistic regression analysis were reported as Odds Ratios (ORs) and 95% Confidence Intervals (CIs) (13). SPSS version 22.0 was used for statistical analyses (IBM Corp. Released 2013. IBM SPSS Statistics for Windows, Version 22.0. Armonk, NY: IBM Corp.)

## RESULTS

**Figure 1:**
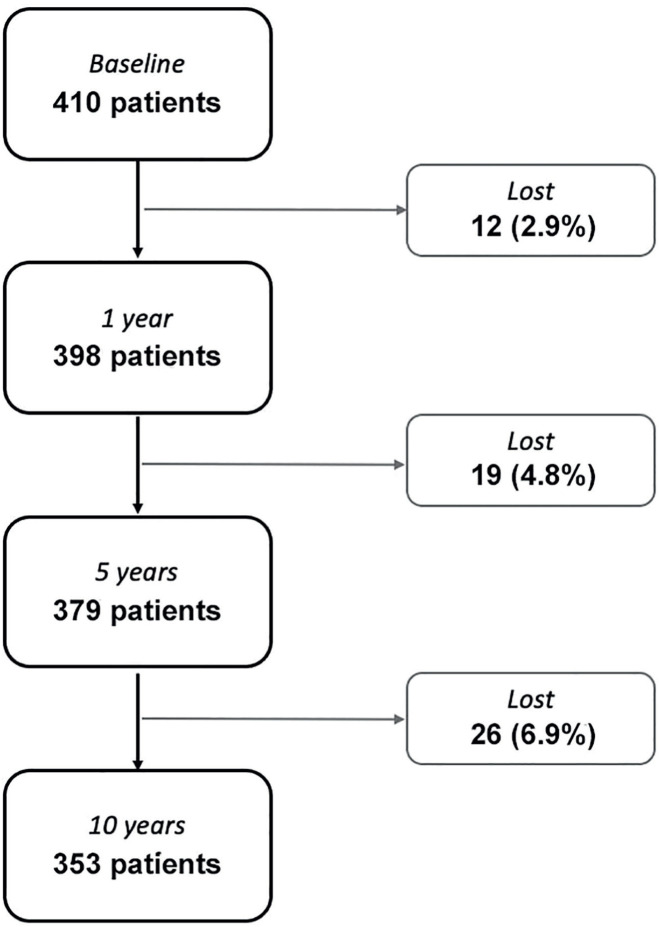
Patients lost to follow-up.

A total of 410 patients with a mean±SD age of 63.9±9.7 years and a mean±SD PV of 115.6±28.6 mL was included in the study. The baseline patient characteristics are reported in Table-1. The mean±SD follow-up was 108.2±29.6 months. Overall, 57 (13.9%) patients were lost during the 10-year follow-up (untraceable: 31; data on primary and secondary outcomes not collected at follow-up: 19, change of medical center: 5; died from other causes: 2), as shown in Figure-1.

**Table 1:** Baseline patient characteristics.

Patients (*n*)		410
**Age**, years *Mean (SD)*		63.9 (9.7)
**BMI** *Mean (SD)*		25.8 (4.6)
**Diabetes mellitus** *n (%)*		63 (15.4)
**Anticoagulants or antiplatelet agents** *n (%)*		51 (12.4)
**PV**, mL *Mean (SD)*		115.6 (28.6)
**PSA**, ng/mL *Mean (SD)*		3.8 (2.8)
**Indwelling bladder catheter** *n (%)*		41 (10)
**BPH medications** *n (%)*		
	α-blockers	236 (57.6)
	5ARI	20 (4.9)
	α-blockers + 5ARI	104 (25.4)
	Others*	9 (2.2)
**IPSS-Total**, points *Mean (SD)*		23.3 (4.7)
**IPSS-QoL**, points *Mean (SD)*		4.0 (0.8)
**Qmax**, mL/s *Mean (SD)*		7.9 (3.9)
**Qave**, mL/s *Mean (SD)*		4.2 (1.4)
**PVR**, mL *Mean (SD)*		127.2 (44.1)

5ARI = 5-Alpha Reductase Inhibitors; BPH = Benign Prostatic Hyperplasia; BMI = Body Mass Index; IPSS = International Prostatism Symptom Score; PSA = Prostate-Specific Antigen; PV = Prostate Volume; PVR = Post-void residual volume; Qave = average urinary flow rate; Qmax = maximum urinary flow rate; QoL = Quality of Life; SD = Standard Deviation.

* Antimuscarinics, **β**3-agonists, or daily Tadalafil 5 mg, not associated with previous BPH drugs.

Mean±SD PV was significantly reduced at 1 year compared to baseline (115.6±28.6 vs. 49.4±12.4 mL; p<0.001). A non-significant increase 5 years (48.2±11.8 vs. 55.3±11.3 mL; p=0.208), and a further significant increase 10 years after surgery (56.9±10.9 vs. 75.1±10.0 mL; p=0.012) in terms of PV was observed. With respect to the previous time point, mean±SD IPSS-Total score was significantly improved 1 year post-operatively (23.3±4.7 vs. 10.3±3.8 points; p<0.001), comparable after 5 years (10.5±3.6 vs. 10.7±5.0 points; p=0.161), and significantly worse 10 years after surgery (10.3±4.8 vs. 13.8±4.5 points; p=0.042), but still statistically and clinically superior to baseline (13.8±4.5 vs. 22.1±4.3 points; p<0.001). In the overall cohort, 349/353 (98.9%) patients reported a MCID in IPSS-Total score in favor of 10-year follow-up compared to baseline. According to PGI-I 10 years after surgery, 317/353 (89.8%) men reported a "much better" or "very much better" overall urinary function compared to their baseline. A comprehensive description of long-term functional outcomes is provided in Table-2.

**Table 2:** Long-term outcomes of ThuLEP.

	Baseline (n=410)	1 year (n=398)	5 years (n=379)	10 years (n=353)	P-values*
**PV**, mL *Mean (SD)*	115.6 (28.6)	49.4 (12.4)	55.3 (11.3)	75.1 (10.0)	**< 0.001^a^** 0.208^b^ **0.012^c^** **< 0.001**^d^
**IPSS-Total**, points *Mean (SD)*	23.3 (4.7)	10.3 (3.8)	10.7 (5.0)	13.8 (4.5)	**< 0.001^a^** 0.161^b^ **0.042^c^** **< 0.001**^d^
IPSS-Total MCID** *n (%)*	NA	394 (99.0)	376 (99.2)	349 (98.9)	0.589^b^ 0.388^c^
**IPSS-QoL**, points *Mean (SD)*	4.0 (0.8)	1.1 (0.9)	1.2 (1.1)	1.6 (1.3)	**< 0.001^a^** 0.327^b^ **0.028^c^** **0.013**^d^
IPSS-QoL MCID** *n (%)*	NA	394 (99.0)	363 (95.8)	319 (90.4)	0.430^b^ 0.134^c^
**Qmax**, mL/s *Mean (SD)*	7.9 (3.9)	21.6 (4.3)	19.0 (3.6)	17.5 (2.6)	**< 0.001^a^** 0.22^b^ 0.38^c^ **< 0.001**^d^
Qmax MCID** *n (%)*	NA	396 (99.5)	371 (97.9)	313 (88.7)	0.289^b^ 0.069^c^
**Qave**, mL/s *Mean (SD)*	4.2 (1.4)	10.9 (2.2)	10.5 (2.9)	10.2 (2.4)	**< 0.001^a^** 0.253^b^ 0.335^c^ **< 0.001**^d^
**PVR**, mL *Mean (SD)*	127.2 (44.1)	50.2 (12.1)	41.0 (23.4)	45.7 (17.5)	**< 0.001^a^** 0.114^b^ 0.249^c^ **< 0.001**^d^
PGI-I of urinary function*** *n (%)*	NA	369 (92.7)	342 (90.2)	317 (89.8)	0.392^b^ 0.183^c^
BPH medical therapy**** *n (%)*	369 (90.0)	2 (0.5)	21 (5.5)	34 (9.6)	**< 0.001^a^** **0.012^b^** 0.059^c^ **< 0.001**^d^
BPH surgical retreatment**** *n (%)*	NA	4 (1.0)	10 (2.6)	21 (5.9)	0.153^b^ 0.065^c^

BPH = Benign Prostatic Hyperplasia; IPSS = International Prostatism Symptom Score; MCID = Minimal Clinically Important Difference; NA = Not Available; PGI-I = Patient Global Impression of Improvement; PV = Prostate Volume; PVR = Post-void residual volume; Qave = average urinary flow rate; Qmax = maximum urinary flow rate; QoL = Quality of Life; SD = Standard Deviation; ThuLEP = Thulium Enucleation of the Prostate.

* In each comparison the mean value at time 1 (pre) was recalculated taking into account only the residual population at time 2 (post); statistically significant p-values were shown in bold.

** MCID cut-offs: IPSS-Total −3 points, IPSS-QoL −0.5 points, Qmax +2 mL/s (compared to baseline).

*** "Much better" or "Very much better"

**Figure 2:**
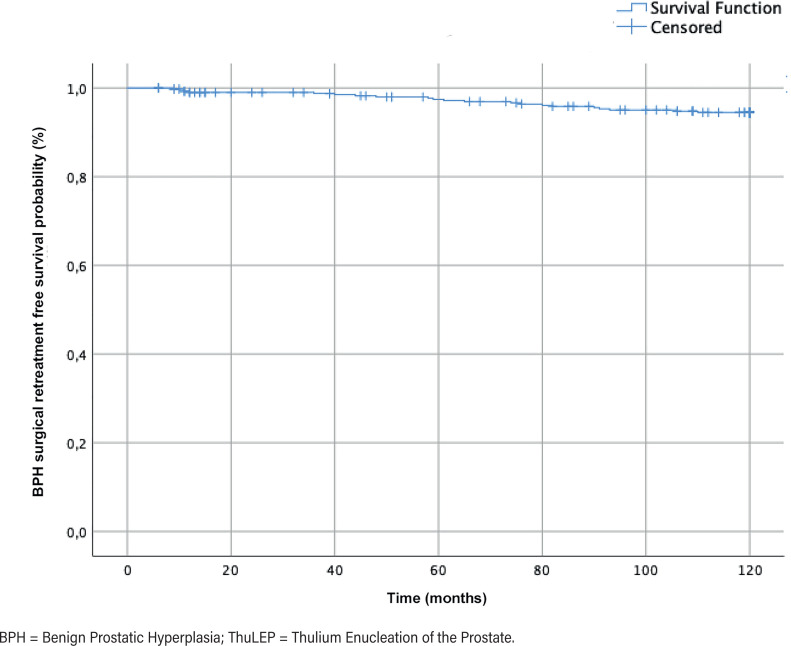
Kaplan-Meier curve: time to BPH surgical retreatment after ThuLEP.

Ten years after ThuLEP, 34/353 (9.6%) patients had restarted BPH pharmacological therapy, while 21/353 (5.9 %) men had undergone BPH surgical retreatment with a mean±SD time to resurgery of 59.8±31.6 months (Figure-2). No episodes of bladder catheterization occurred during the follow-up. According to the multivariable analysis, only baseline PV (adjusted OR 1.27, 95% CI 1.09-1.41; p<0.001) and time from BPH surgery (adjusted OR 1.32, 95% CI 1.15-1.43; p<0.001) were detected as predictors of BPH surgical retreatment (Table-3).

**Table 3:** Predictors of BPH surgical retreatment 10 years after ThuLEP.

		Multivariable model		
		OR	95% CI	P-value*
**Age**, *years*		1.04	0.96-1.08	0.167
**Baseline PV**, *mL*		1.27	1.09-1.41	<0.001
**Time from BPH surgery**, *years*		1.32	1.15-1.43	<0.001
BPH medical therapy after surgery				
	No	Reference	Reference	-
	Yes	1.15	0.96-1.27	0.089

BPH = Benign Prostatic Hyperplasia; CI = Confidence Interval; OR: Odds Ratio; PV = Prostate Volume; ThuLEP = Thulium Enucleation of the Prostate.

* Statistically significant p-values were shown in bold.

Logistic regression analysis was used to detect the predictors of BPH surgical retreatment.

Age, baseline PV, BPH medical retreatment, and time from BPH surgery were selected a priori as independent variables.

## DISCUSSION

Surgery is an effective treatment for BPH-related LUTS. It does not involve removal of the entire prostate, but focuses only on the hyperplastic transitional zone, to reduce the risk of serious complications (14). However, it exposes patients to the risk of regrowth of prostatic tissue, worsening of LUTS, and possible need for medical or surgical retreatment (15). When enough hyperplastic tissue is removed during surgery, the prostate takes several years to redevelop and impair urinary function; therefore, only long-term studies can reliably evaluate the functional outcomes of BPH surgical treatment. In addition to time from surgery, several other factors could influence the risk of LUTS recurrence and BPH retreatment (e.g., type of surgical procedure, surgeon experience, patient age, and PV); however, the evidence available on the topic is limited (5, 16–19).

In our cohort of 410 patients undergoing ThuLEP, we found a BPH surgical retreatment rate of 5.9% after 10 years; in addition, time from BPH surgery and PV were identified as possible predictors of surgical retreatment. The relationship between prostate size and negative BPH outcomes has been established for many years (20). In our series, significant enlargement of the prostate between 5 and 10 years of follow-up coincided with significant worsening of LUTS in the same period. In addition, PV at baseline was a significant predictor of BPH reoperation, probably due to increased surgical complexity with suboptimal tissue removal or faster tissue regrowth after surgery as volume increases.

Only one study on long-term outcomes is available for comparison with our findings. Grüne et al. described the outcomes of 1,097 patients with a median PV of 90 mL who underwent ThuLEP (5). This retrospective single-center study had a median follow-up period of 72 months. A total of 42 (3.8%) men underwent BPH surgical retreatment, and most of them (33, 78.6%) underwent surgery within 5 years of the first surgery (median: 24 months). On multivariate analysis, only enucleation weight ≥ 60 g was identified as a predictor of surgical retreatment (Hazard Ratio 1.19, 95% CI 1.03-1.36; p=0.014). Our study confirms most of Grüne's findings, considering a longer follow-up period and a smaller but still considerable number of patients (5). Another term of comparison could be found in the limited number of long-term studies on HoLEP. Elmansy et al. reported the outcomes of 949 patients with a mean PV of 81 mL undergoing HoLEP (21). The authors conducted a retrospective single-center analysis with a mean follow-up of 62 months; however, only 89 (9.4%) men completed 10 years of follow-up. All functional outcomes (IPSS, QoL, Qmax, and PVR) showed significant improvements 1 month after surgery and throughout the study period. At 10-year follow-up, mean IPSS, QoL, Qmax, and PVR were 3.6 points, 0.7 points, 26.9 mL/s, and 20.7 mL, respectively. The BPH reoperation rate was 0.7%. Fallara et al. described the outcomes of 125 patients with a median PV of 78 mL undergoing HoLEP (22). This was a retrospective single-center study with a median follow-up period of 126 months. According to the authors’ criteria (IPSS < 8, Qmax > 15 mL/s, PVR < 20 mL, no resurgery for bladder outlet obstruction, no need for medications for LUTS), 74% of the patients were asymptomatic at long-term follow-up. At 126 months, the median IPSS, Qmax, and PVR were 5 points, 16 mL/s, and 10 mL. None of the patients underwent surgical retreatment for prostate regrowth, while 3 (2.4%) men required medications for LUTS. No long-term comparison between ThuLEP and HoLEP, ThuLEP and simple prostatectomy, or ThuLEP and minimally invasive surgical procedures for BPH is available in literature.

To the best of our knowledge, this is currently the study available on ThuLEP with the longest follow-up. The use of validated tools for the evaluation of functional outcomes, the relatively large number of patients included, and the low data loss to follow-up are further strengths of our research. However, some limitations must be considered when interpreting our results. First, the retrospective design is associated with a high risk of bias, especially for long follow-up periods. In addition, the single-center single-surgeon design may limit the generalizability of our findings. Moreover, numerous clinical characteristics of the patients may have influenced the outcomes, and only some of them were collected. Finally, the surgical outcomes, perioperative data, and complications were not recorded. In particular, the occurrence of urethral strictures, bladder neck sclerosis, urinary incontinence, and related treatments were not reported during follow-up. These factors may have influenced the reported outcomes.

In conclusion, the optimal results achieved with ThuLEP appear to be sustained during long-term follow-up. Ten years after the primary treatment, LUTS were significantly improved compared to the baseline, and there was a low incidence of BPH surgical retreatment throughout the study period. Notably, the baseline PV and the time from surgery were identified as significant predictors of the need for surgical retreatment.

## Data Availability

The database and the raw results of the data analysis are available upon reasonable request to the corresponding author.
